# High Performance MXene/MnCo_2_O_4_ Supercapacitor Device for Powering Small Robotics

**DOI:** 10.1021/acsaelm.4c01204

**Published:** 2024-09-19

**Authors:** Nanasaheb M. Shinde, Martin Pumera

**Affiliations:** Advanced Nanorobots & Multiscale Robotics Laboratory, Faculty of Electrical Engineering and Computer Science, VSB - Technical University of Ostrava, 17. listopadu 2172/15, 70800 Ostrava, Czech Republic

**Keywords:** 2D materials, nanocomposite, electrodeposition, supercapacitors, energy storage, portable electronics

## Abstract

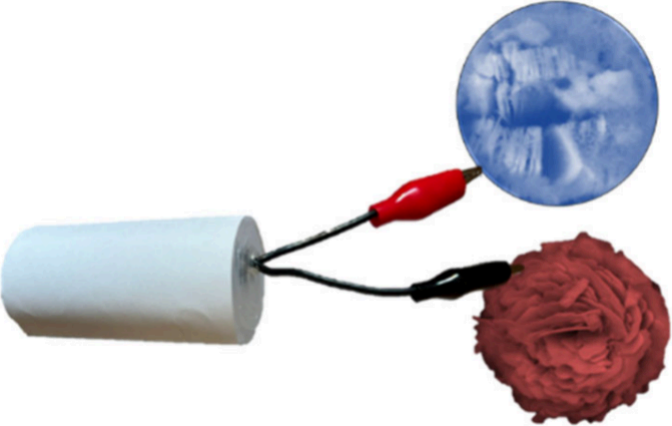

The development of advanced energy storage devices is critical for various applications including robotics and portable electronics. The energy storage field faces significant challenges in designing devices that can operate effectively for extended periods while maintaining exceptional electrochemical performance. Supercapacitors, which bridge the gap between batteries and conventional capacitors, offer a promising solution due to their high power density and rapid charge–discharge capabilities. This study focuses on the fabrication and evaluation of a MXene/MnCo_2_O_4_ nanocomposite supercapacitor electrode using a simple and cost-effective electrodeposition method on a copper substrate. The MXene/MnCo_2_O_4_ nanocomposite exhibits superior electrochemical properties, including a specific capacitance of 668 F g^–1^, high energy density (35 Wh kg^–1^), and excellent cycling stability (94.6% retention over 5000 cycles). The combination of MXene and MnCo_2_O_4_ enhances the redox activity, electronic conductivity, and structural integrity of the electrode. An asymmetric supercapacitor device, incorporating MXene/MnCo_2_O_4_ as the positive electrode and Bi_2_O_3_ as the negative electrode, demonstrates remarkable performance in powering small robotics and small electronics. This work underscores the potential of MXene-based nanocomposites for high-performance supercapacitor applications, paving the way for future advancements in energy storage technologies.

## Introduction

Today, various energy storage devices including fuel cells, batteries, and supercapacitors are the greatest possible answers to the energy storage device.^1,2^ An ideal supercapacitor device should possess large energy storage capability as well as power density with a long lifecycle.^3,4^ Batteries can store energy for long time have been reported to have advanced energy density and lower power density.^5,6^ Capacitors possess high/low energy/power density, with shorter discharge time.^7,8^ Supercapacitors lie in between conventional capacitors and batteries in terms of energy density and power density.^9,10^ The performance evaluation of the supercapacitor mainly depended on the electrode material used for the fabrication of the supercapacitor device. To achieve this, there is continuous hunting for nanocomposites having higher energy density and longer stability with highly porous nanostructures due to their characteristics and good performance in a broad range of applications.^11,12^ MXene, a promising 2D material, is a broad collection of transition metal carbides, nitrides, and carbonitrides.^13,14^ MXene exhibits very good structural, electrical, and electrochemical characteristics.^15,16^ The basic chemical structure is M_*y+*1_X_*y*_T_*x*_, where M = transition metal, X = C/N/C_*x*_N_*y*_, and T = −OH, −O, −F, etc.^17^ MXene has a high surface area and variable oxidation states, which is an advantageous application in supercapacitors.^18^ Although pristine MXene offers outstanding electrochemical properties when it is employed for real commercial applications for a longer period, it faces some issues; i.e., MXene experiences restacking of nanosheet structures.^19^ Therefore, direct use of 2D MXene has limitations including lower capacitance, reduced stability, a nonreactive nanostructure, and hindered electrolyte diffusion due to restacking.^20,21^ MXene has a high electronic conductivity and, when combined with other transition metal oxide materials, increases the electrochemical performance of supercapacitor devices.^22−25^ The MXene and transition metal oxide nanocomposite have received growing research because of having active redox sites, numerous oxidation states, and excellent electric conductivity.^26−31^ With this inspiration, there are few reports available on binary transitional metal oxides, and the MXene nanocomposite has been reported for supercapacitor application^32−36^ and obtained electrochemical performance with lower stability and capacitance,^37,38^ which is still not sufficient for making a supercapacitor device for practical applications. Also, the prepared MXene nanocomposite electrode is in the form of a powder and requires another polymer (such as activated carbon, poly(vinylidene fluoride), polytetrafluoroethylene, etc.) for making supercapacitor electrodes and prolonged time-consuming synthesis steps.^39,40^ To overcome this scientific issue and improve electrochemical performance, we have used electrodeposition as simple, cost-effective, and fast growth binder-free method to fabricate MXene/MnCo_2_O_4_ electrodes on a copper substrate.

**Scheme 1:**
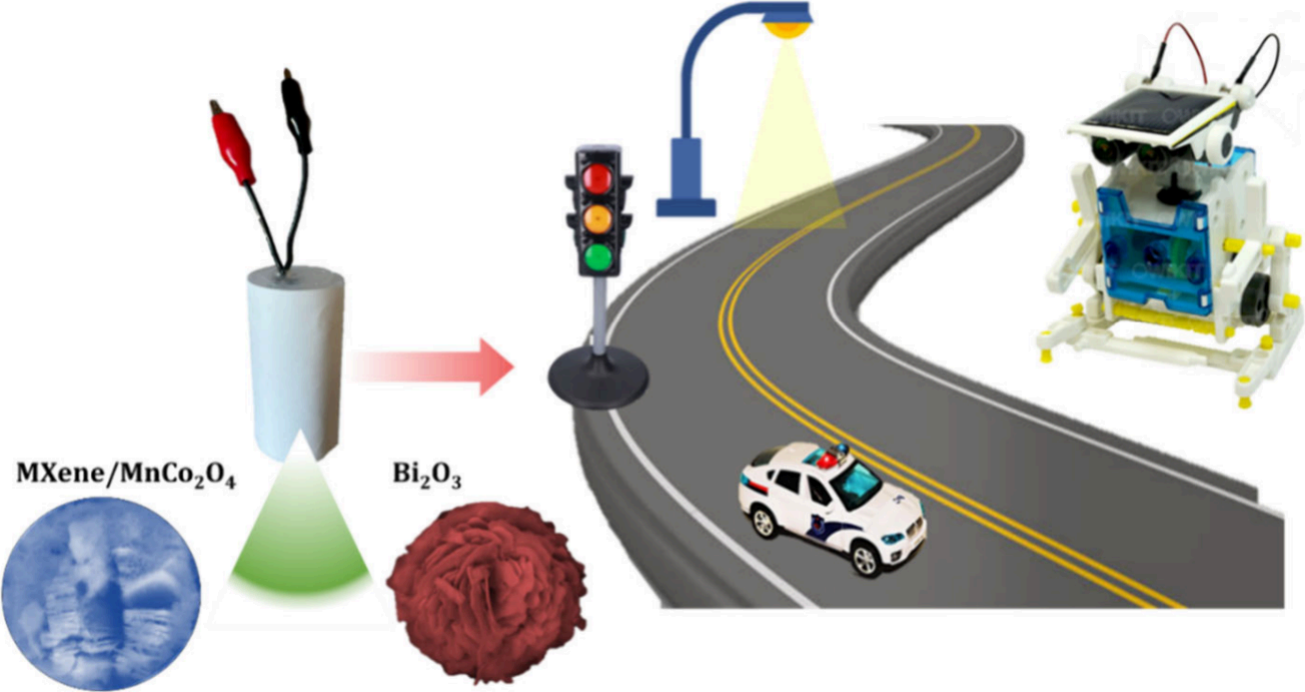
Schematic Concept for MXene/MnCo_2_O_4_ Nanocomposite Prototype Supercapacitor Device to Powering a Small Robot and Small Electronics

In this work, herein, we present an MXene/MnCo_2_O_4_ supercapacitor electrode on the copper substrate through electrodeposition deposition (Scheme 1), followed by a prototype supercapacitor device fabricated using the MXene/MnCo_2_O_4_ as positive and Bi_2_O_3_ as negative electrode. As a proof-of-concept, the fabricated supercapacitor device demonstrated a remarkable electrochemical performance with powering small robotics, lighting red/blue LED, toy traffic signal light, and lighting of toy police car signals, signifying their potential for high-performance energy storage device applications (Scheme 1).

## Results and Discussion

**Figure 1:**
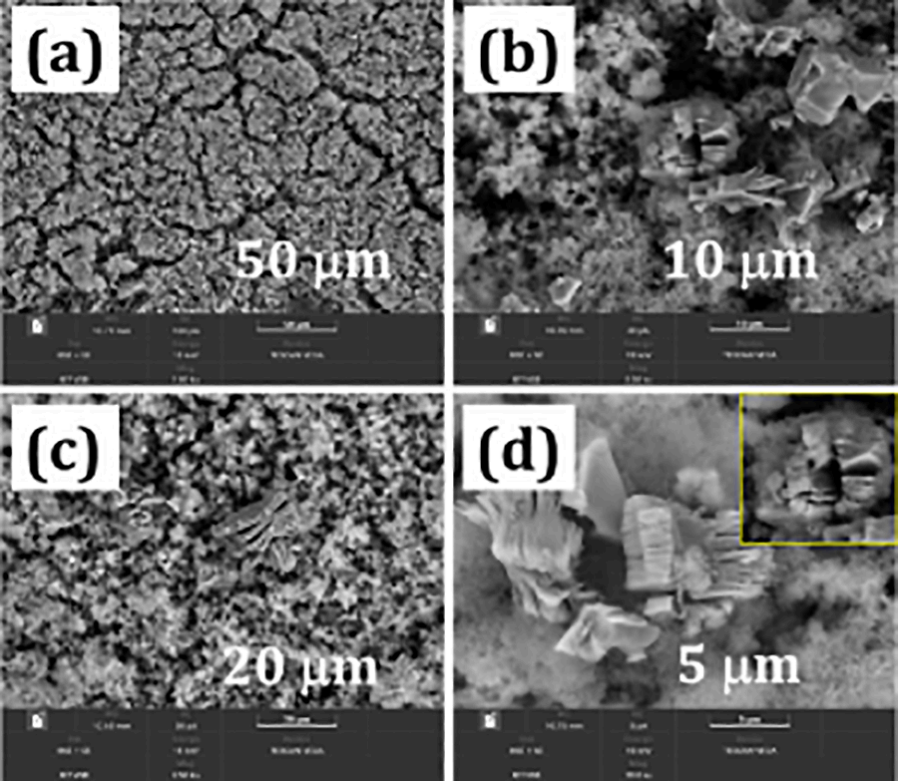
(a–d) FE-SEM images of the MXene/MnCo_2_O_4_ electrode (inset in panel d shows a zoomed-in FE-SEM view of MXene/MnCo_2_O_4_).

**Figure 2:**
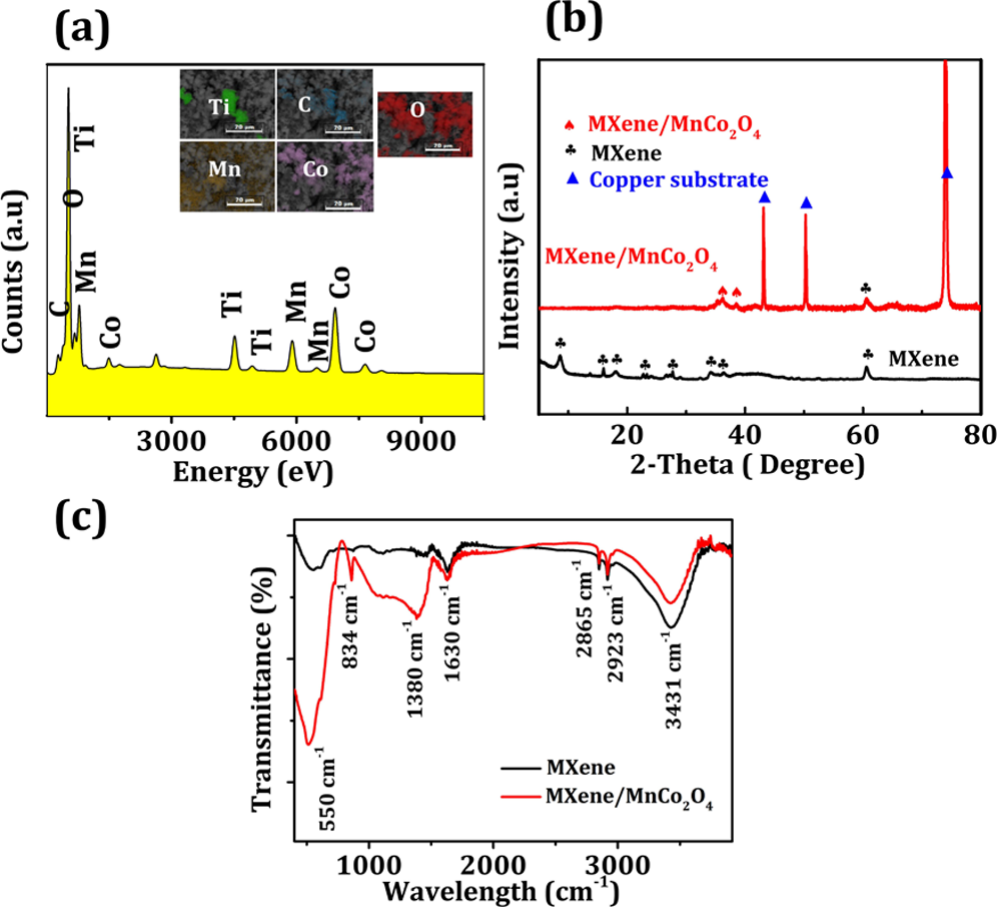
(a) EDX spectra (inset shows Ti, Mn, Co, and O elements color mapping) of MXene/MnCo_2_O_4_ electrode. (b) XRD pattern and (c) FT-IR spectra of MXene and MXene/MnCo_2_O_4_ electrodes.

The growth of MnCo_2_O_4_ nanoparticles over MXene nanosheets preserves agglomeration and improves supercapacitor performance. FE-SEM image of MXene/MnCo_2_O_4_ shows the rough surface and stacked layered nanostructure of MXene (see also images in Figure S1) coated with MnCo_2_O_4_ nanoparticles (Figure 1a–d). During the electrodeposition process, the Co and Mn ions migrated to the interior part of the MXene and, as a result, coated MnCo_2_O_4_ nanoparticles over MXene nanosheet with an enlarged volume and more redox active sites, for fast electrolyte ion adsorption and desorption process. The FE-SEM cross-section images revealed that the average thickness of the prepared MXene/MnCo_2_O_4_ electrode is approximately 12 μm, while the MXene electrode alone has a thickness of around 10 μm (see Figure S2a,b). The EDX spectra shown in Figure 2a demonstrate the presence of Ti, C, Mn, Co, and O significant peaks, confirming the effective synthesis of MXene/MnCo_2_O_4_ nanocomposite. The inset of Figure 2a displays the SEM-EDX image of the MXene/MnCo_2_O_4_ nanocomposite exhibiting the distribution of Ti, Co, Mn, O, and C. The results show that MXene/MnCo_2_O_4_ nanocomposite contains 17.36 wt % of C, 44.43 wt % of O, 5.51 wt % of Ti, 7.04 wt % of Mn, and 17.66 wt % of Co. Whereas, trace amounts of 0.81 wt % of Al, 5.64 wt % of nitrogen, and 1.56 wt % of Cl confirm the successful synthesis of MXene from the MAX phase. The corresponding elemental composition of the MXene/MnCo_2_O_4_ nanocomposite is listed in Table S1. From this analysis we gave an essential remark that the MXene/MnCo_2_O_4_ nanocomposite was effectively synthesized. Figure 2b shows the XRD patterns of MXene and MXene/MnCo_2_O_4_ nanocomposite MXene composites. The diffraction peaks (marked as black spades) observed at 8.48°, 18.59°, 34.0°, 36.52°, and 60.76° correspond to the MXene. Additionally, the appearances of less intense peaks (marked as black spades) at 15.86°, 23.52°, and 27.74° were attributed to the presence of TiO_2_.^35−40^ This is due to the Ti and F elements in the Ti_3_C_2_T_*x*_ (MXene) oxidated during the etching process or may be due to oxidation; this was problematic to eliminate, in agreement with a previously reported paper elsewhere.^41−43^ In the XRD patterns of MXene/MnCo_2_O_4_, peaks reflected (marked as black spades) at 36.2° and 38.3°of MnCo_2_O_4_ (PDF# 84-0482). In addition, the MXene peak was detected with a reduced intensity around 60.76°, implying that the MnCo_2_O_4_ nanoparticles were successfully grown over the surface of MXene nanosheets. Upon closer inspection of XRD spectra of MXene/MnCo_2_O_4_ nanocomposites, the reason for the vanishing XRD peaks of MXene is because of the partial oxidation and inhibited agglomeration of MXene nanosheets by randomly decorating MnCo_2_O_4_ metal oxide nanoparticles, which is well consistent with the reflected FE-SEM result shown in Figure 1a–d. Whereas, the higher peaks marked with ▲ belonged to the copper substrate (see Figure 2b), which could be responsible for suppressing the characteristics peak of MXene/MnCo_2_O_4_ nanocomposites and the pristine MXene electrode. The XRD analysis confirmed the formation of binder-free MXene/MnCo_2_O_4_ nanocomposites on a copper substrate, which is consistent with the FE-SEM result. Structural and morphology result analysis illustrates the binder-free electrodeposition syntheses of MXene/MnCo_2_O_4_ nanocomposites on a copper substrate.

The surface functionality of the MXene/MnCo_2_O_4_ and MXene was investigated via FTIR spectroscopy (Figure 2c). The spectra of MXene/MnCo_2_O_4_ and MXene exhibited characteristic bands at 550, 834, 1380, and 1630 cm^–1^ corresponding to Ti–O, CO_3_^2–^, O–H, and C=O, respectively, showing that MnCo_2_O_4_ has been successfully grown on the surface of MXene.^44,45^ In addition, the presence of hydroxyl groups was confirmed by the absorption peaks at 3431 cm^–1^, which were assigned to the absorbed external water and hydrogen-bonded OH^–^ in the MXene/MnCo_2_O_4_ and MXene electrode. The strong absorption peaks at 2923 and 2865 cm^–1^ corresponded to stretching vibrations of the C–H and–CH_2_ groups, respectively.^46−48^ The existence of these groups in the MXene/MnCo_2_O_4_ and MXene indicates the formation of the successful deposition of MnCo_2_O_4_ on the MXene.

**Figure 3:**
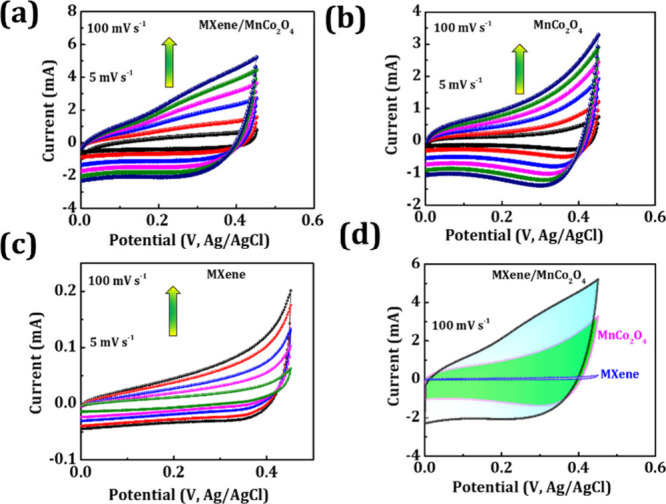
(a–c) Cyclic voltammograms of MXene/MnCo_2_O_4_, MnCo_2_O_4_, and MXene electrodes at different scan rates of 5–100 mV s^–1^ and (d) their comparative cyclic voltammograms at a scan rate of 100 mV s^–1^.

The half-cell electrochemical analyses such as cyclic voltammetry, galvanostatic charge–discharge, and electrochemical impedance spectroscopy of the MXene/MnCo_2_O_4_ nanocomposite, MXene, and MnCo_2_O_4_ have been carried in 6 M KOH electrolyte, with a Pt plate counter electrode, Ag/AgCl reference elecrode, and MXene/MnCo_2_O_4_, MXene, and MnCo_2_O_4_ working electrodes. Figure 3a–d exhibits the cyclic voltammograms of the MXene/MnCo_2_O_4_ nanocomposite, MXene, and MnCo_2_O_4_ in the potential window (scan rates) 0.45–0 V (5–100 mV s^–1^). The cyclic voltammetry curves of the MXene/MnCo_2_O_4_ nanocomposite, MnCo_2_O_4_, and MXene exhibit a pseudocapacitive shape at different scan rates due to the electrolyte ions adsorption/desorption on the surface.^31,32^ As seen from Figure 3a–d, with increased scan electrodes, the area under the cyclic voltammetry curve of prepared electrodes was increased systematically without disturbing the shape of the curve significantly, which specifies that the prepared electrodes have good electrochemical reversibility.^33−35^ Upon comparison of the cyclic voltammetry curve profile as shown in Figure 3d, the MXene/MnCo_2_O_4_ nanocomposite displays a bigger integral cyclic voltammetry curve area because of the combination consequence between MnCo_2_O_4_ and MXene. Cyclic voltammetry curves indicate that the MXene/MnCo_2_O_4_ nanocomposite exhibits stable pseudocapacitive behavior. The pseudocapacitive nature of the MXene/MnCo_2_O_4_ composite was further confirmed through X-ray photoelectron spectroscopy (XPS) analysis after thousands of repeated cyclic voltammetry cycle scans. The presence of Mn, Co, O, Ti, and C elements in the MXene/MnCo_2_O_4_ composite was confirmed by the survey spectrum (Figure S3). From XPS analysis, the MXene/MnCo_2_O_4_ composite indicated that individual element peaks were reflected at the expected position without shift in peak position, implying the higher stability MXene/MnCo_2_O_4_ during cycling mechanisms.^32−34,48,49^ The corresponding XPS of individual MnCo_2_O_4_ and MXene electrodes are shown in Figures S4 and S5.

**Figure 4:**
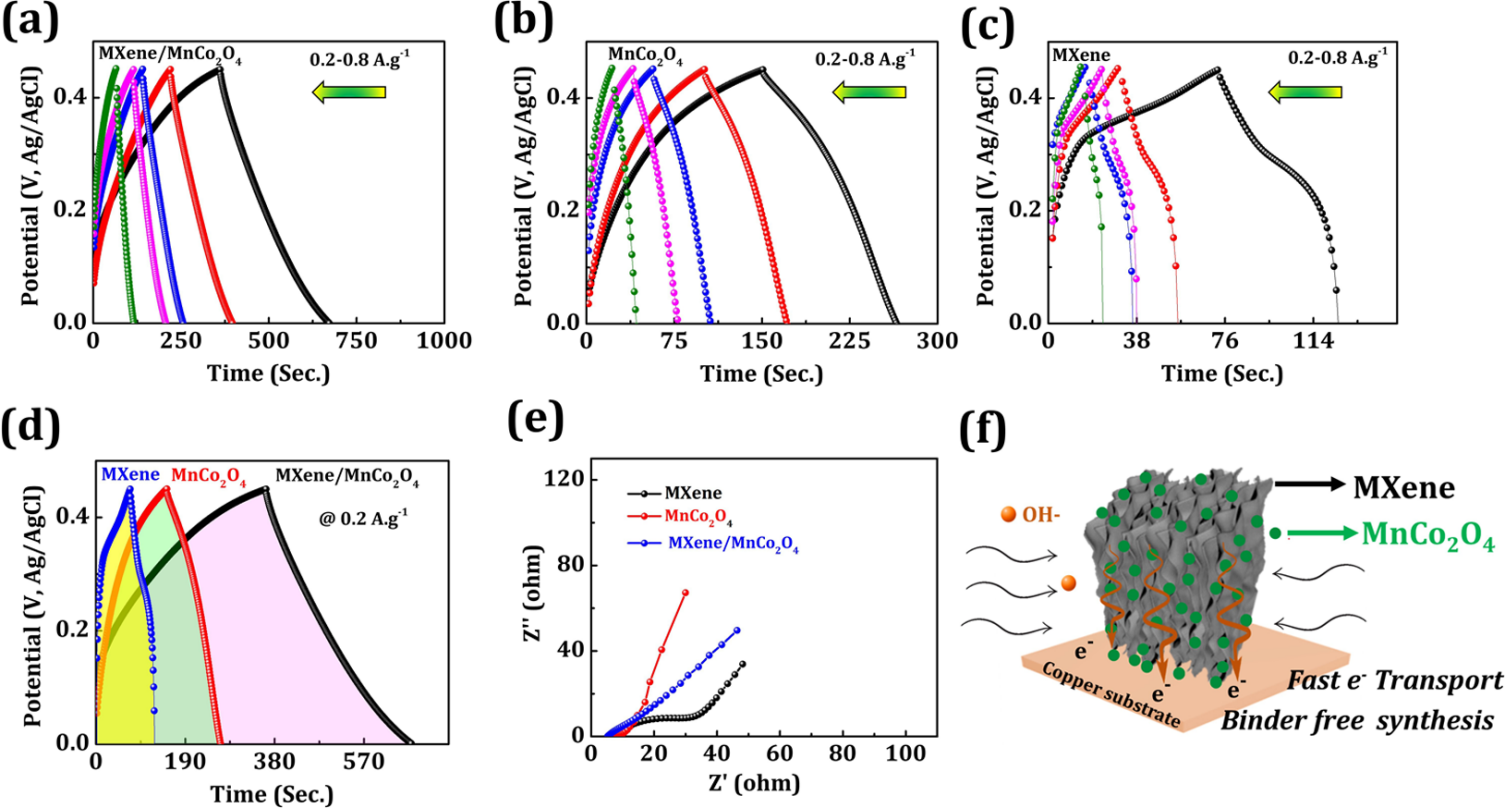
(a–c) Galvanostatic charge–discharge curves of MXene/MnCo_2_O_4_, MnCo_2_O_4_, and MXene electrodes at different applied current densities (0.2–0.8 A g^–1^) and (d) their comparative galvanostatic charge–discharge curves at a fixed scan rate of 0.2 A g^–1^. (e) EIS spectrum of MXene/MnCo_2_O_4_, MnCo_2_O_4_, and MXene electrodes. (f) Schematic illustration for ion charge process mechanism in the MXene/MnCo_2_O_4_ nanocomposite.

Figure 4a–d shows the galvanostatic charge–discharge profile of the MXene/MnCo_2_O_4_ nanocomposite, MXene, and MnCo_2_O_4_ electrode at different applied current densities at 0.2–0.8 A g^–1^. The galvanostatic charge–discharge profiles of MXene, MnCo_2_O_4_, and MXene/MnCo_2_O_4_ nanocomposites exhibiting deviation from the perfect triangular shape of the galvanostatic charge–discharge curve of MXene/MnCo_2_O_4_ nanocomposites exhibit a pseudocapacitance behavior due to redox reactions.^34,35^ Upon comparison of galvanostatic charge–discharge curves (see Figure 4d) of MXene and MnCo_2_O_4_ electrodes, the MXene/MnCo_2_O_4_ nanocomposites have elongated charge–discharge times and display greater specific capacitance. The specific capacitance values of MXene/MnCo_2_O_4_ nanocomposites, MnCo_2_O_4_ and MXene, electrodes were obtained approximately 668–443, 187–89, and 90–46 F g^–1^ at applied current density 0.2–0.8 A g^–1^, respectively (see the calculation details in Section S2 of the Supporting Information). To further investigate the ion transportation kinetics, the Nyquist plots of MXene/MnCo_2_O_4_ nanocomposites, MXene, and MnCo_2_O_4_ electrodes were analyzed and are presented in Figure 4e. With this assumption, the diameter of the semicircle display at a high frequency represents the charge transfer resistance (*R*_ct_) between the electrode and electrolyte. Figure 4e shows that the MXene/MnCo_2_O_4_ nanocomposites have charge transfer resistances (∼2 Ω), and it has a minor semicircular diameter compared to MXene (∼27 Ω) and MnCo_2_O_4_ (∼8 Ω).^36−38^ In addition, the MXene/MnCo_2_O_4_ nanocomposite electrode displayed excellent cycling stability at around 90.6% at 5000 continuous galvanostatic charge–discharge cycles compared to MXene and MnCo_2_O_4_ electrode (Figure S6). The study observed a slight decrease in the stability of the MXene/MnCo_2_O_4_ nanocomposite after 5000 cycles. This decrease in stability was attributed to the peeling off of the MXene/MnCo_2_O_4_ nanocomposite material. Figure S7 shows FE-SEM images of the electrode after these 5000 cycles, revealing that the 2D nanosheets of MXene were significantly changed in morphology. The changes are related to the formation of aggregated particles, which exhibit a morphology different from that of the original nanostructure.

**Figure 5:**
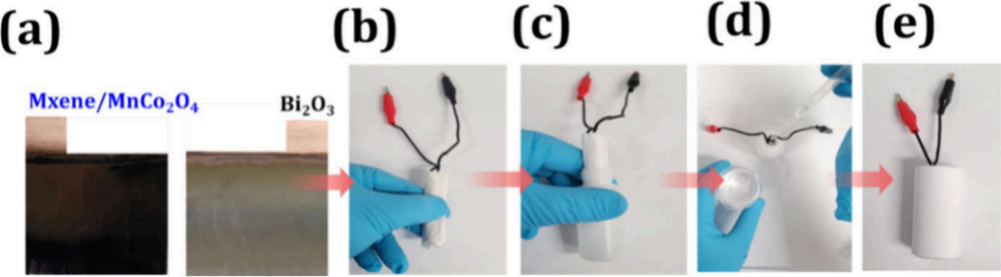
Steps involving fabrication of MXene/MnCo_2_O_4_ asymmetric devices. (a) Photographs of MXene/MnCo_2_O_4_ (right) and Bi_2_O_3_ (left) electrodes deposited on (4 × 3 cm^2^) large-area copper substrates. (b) Electrical contacts. (c, d) Folding and both electrodes immersed in the 6 M KOH electrolyte in a plastic container. (e) Actual digital photograph of laboratory scale fabricated MXene/MnCo_2_O_4_//Bi_2_O_3_ supercapacitor devices.

The above-mentioned result indicates that the synthesis of the MXene/MnCo_2_O_4_ electrode shows higher or comparable specific capacitance than other MXene nanocomposite electrode materials reported previously (see Table S2),^32−36,50−52^ which may be due to binder-free synthesis of MXene and MnCo_2_O_4_ oxide nanocomposite enhancing the supercapacitor performance; the well-decorated MnCo_2_O_4_ over the conductive MXene surface acts as superhighway track for electron transport as shown in Figure 4f. Finally, the adhesion of MXene/MnCo_2_O_4_ nanocomposite nanostructures with the base copper substrate via direct coating using electrodeposition makes this electrode mechanically stable. Electrodeposition allows for fast electron transport of charges/ions from the MXene/MnCo_2_O_4_ nanocomposite to the copper substrate. Uniting these nanocomposite features can be used to fabricate high-performance supercapacitor devices for powering small robotics, lighting red/blue LED, toy traffic signal light, and lighting of a police car signal. In order to exploit the MXene/MnCo_2_O_4_ electrode, an asymmetric supercapacitor device with MXene/MnCo_2_O_4_ and Bi_2_O_3_ as positive and negative was fabricated (see Figure 5a). The detailed experimental, FE-SEM, and electrochemical performance of the Bi_2_O_3_ electrode have been added in Figures S8–S10. For asymmetric supercapacitor device fabrication, an identical large size (4 × 3 cm^2^) MXene/MnCo_2_O_4_ and Bi_2_O_3_ electrodes were deposited on a copper substrate. Then, these two electrodes were wound around each other to form a roll and then placed into a plastics tube (purchased from local Czech bazaar) which was filled with 6 M KOH electrolyte, as shown in Figure 5a–e.

**Figure 6:**
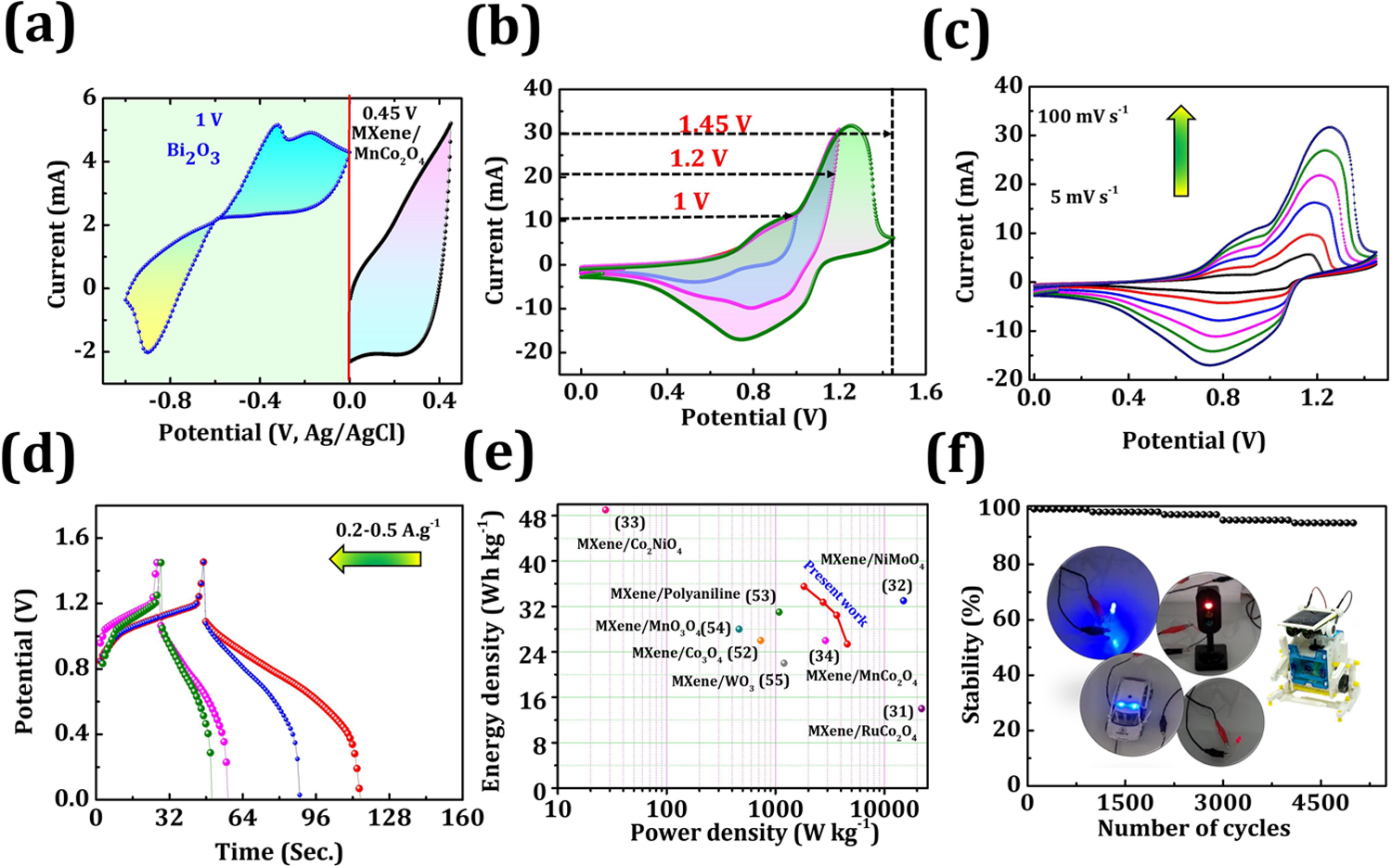
Comparison of cyclic voltammograms of (a) Bi_2_O_3_ and MXene/MnCo_2_O_4_ electrodes at a fixed scan rate of 100 mV s^–1^. (b) Cyclic voltammetry curves for different voltages (1–1.45 V) at a fixed scan rate 100 mV s^–1^. (c) Cyclic voltammetry curves at various scan rates (5–100 mV s^–1^) at 1.45 V. (d) Galvanostatic charge–discharge curves. (e) Ragone plots compared to recently reported supercapacitor devices and (f) stability of the MXene/MnCo_2_O_4_//Bi_2_O_3_ (inset of part f shows a photograph of the power-up toy traffic light, red/blue LED, lighting of toy police cars signal, and small robotics).

Wherein, an insulating paper separator was placed between MXene/MnCo_2_O_4_ and Bi_2_O_3_ electrodes, in order to avoid a short circuit of the supercapacitor device. In the end, the wire connection was drawn externally from each electrode; the whole device was completed perfectly closed with the help of glue to avoid leakage of electrolytes from it. The above supercapacitor device fabrication steps are visualized in a digital pictorial in Figure 5a–e. The supercapacitor performance of the MXene/MnCo_2_O_4_//Bi_2_O_3_ asymmetric device was tested and is summarized in Figure 6. Figure 6a shows the cyclic voltammograms of MXene/MnCo_2_O_4_ and Bi_2_O_3_ electrodes, revealing a voltage difference of about 1.45 V. The MXene/MnCo_2_O_4_//Bi_2_O_3_ supercapacitor device established a scientifically stretched voltage range up to 1.45 V (Figure 6b).

The voltage window of the MXene/MnCo_2_O_4_//Bi_2_O_3_ supercapacitor device is elaborated to 1.45 V, which delivers high energy densities because the energy density of the supercapacitor device is proportional to the square of the potential voltage. The cyclic voltammetry analysis of the MXene/MnCo_2_O_4_//Bi_2_O_3_ supercapacitor device at 5–100 mV s^–1^ different scan rates reflected an increased area under the curve, suggesting an excellent rate capability (Figure 6c).^31^ The galvanostatic charge–discharge curves of the MXene/MnCo_2_O_4_//Bi_2_O_3_ supercapacitor device are shown in Figure 6d; the nature of galvanostatic charge–discharge curves is symmetric at different applied current densities, suggesting fabricated supercapacitor device excellent capacitance behavior. Figure 6e is the Ragone plot of the specific energy vs specific power of the MXene/MnCo_2_O_4_//Bi_2_O_3_ supercapacitor device, which reveals the specific energy (specific power) of 35 Wh kg^–1^(1854 W kg^–1^) at the current densities of 1 A g^–1^. Present work shows at several points better performance is superior to those reported previously for the supercapacitor devices listed (Table S3).^31−34,52−55^ Accordingly, the stability of the MXene/MnCo_2_O_4_//Bi_2_O_3_ supercapacitor device recorded a stability of 94.6% over 5000 cycles (at 0.5 A g^–1^), as shown in Figure 6f. In the inset of Figure 6f, the digital image provides evidence for the actual application of the MXene/MnCo_2_O_4_//Bi_2_O_3_ device where powering small robotics, lighting red/blue LED, toy traffic signal light, and lighting of a toy police car signal as an energy storage application for several minutes with lighting at a sufficient brightness intensity (Video S1). Therefore, it is confirmed that the MXene/MnCo_2_O_4_//Bi_2_O_3_ device, as an all-in-one powering energy source, can efficiently power the plenary of a portable electronic device.

## Conclusion

In summary, we proposed a MXene/MnCo_2_O_4_ nanocomposite supercapacitor utilized to power small robotics. The fabricated MXene/MnCo_2_O_4_ nanocomposites exhibited a specific capacitance of 668 F g^–1^. The fabricated supercapacitor device exhibited an energy density of 35 Wh kg^–1^ (power density: 1854 W kg^–1^) with a stability of 94.6%, showing a higher electrochemical performance for potential applications in energy storge devices. The performance of our laboratory scale fabricated supercapacitor device technology was very good. We believe that our laboratory scale fabricated supercapacitor device for future high-performance energy storage devices in the powering portable electronics. We show higher electrochemical performance in the MXene nanocomposite for supercapacitor devices. The present strategy for preparing such MXene nanocomposites will open up opportunities for a wide range of applications beyond energy storage, such as in area of electrocatalysis, gas sensors, or portable water purification.^56^

## Experimental Section

Initially, the copper substrate (proper dimensions 4 × 3 cm^2^) was cleaned with ethanol and distilled water and then dried in an oven at 50 °C for several hours. Further, the MXene/MnCo_2_O_4_ nanocomposite was coated onto the copper substrate from a solution containing 0.1 M Co(NO_3_)_2_ and Mn(NO_3_)_2_ and 10 mg of MXene using the potentiodynamic electrodeposition method in the potential window range 0–1.3 V vs Ag/AgCl at 50 mV s^–1^ scan rate for 50 cycles. After depositing MXene/MnCo_2_O_4_ nanocomposites, electrodes were rinsed with distilled water and then air annealed in the oven at 400 °C for 3 h as shown in Scheme S1. then, fabricated the MXene/MnCo_2_O_4_//Bi_2_O_3_ asymmetric supercapacitor. The various characterization tools were used for structural and electrochemical analysis; corresponding instrumental details have been added in Supporting Information Section S1.

## Supporting Information Available

List of the contents of Supporting Information as formulas used for supercapacitor; materials characterizations techniques; electrochemical and structural analysis of the Bi_2_O_3_ electrode; FE-SEM images of MXene/MnCo_2_O_4_ and Bi_2_O_3_ electrode after 5000 cycles; FE-SEM cross section images of MXene/MnCo_2_O_4,_ MXene and Bi_2_O_3_ electrode; FE-SEM images of MXene electrode; stability of MXene/MnCo_2_O_4,_ MXene and Bi_2_O_3_, specific capacitance, stability, synthesis methods, energy and power density of present work and other recently works (PDF)
Video of powering small robotics, lighting red/blue LED, toy traffic signal light, lighting of toy police cars signal using MXene/MnCo_2_O_4_//Bi_2_O_3_ asymmetric supercapacitor device; chemical composition table of MXene/MnCo_2_O_4_ electrode (MP4)
