# Supplementary material for: High Performance MXene/MnCo2O4 Supercapacitor Device for Powering Small Robotics
Source: ACS Appl Electron Mater. 2024 Sep 19;6(10):7339–45. doi: 10.1021/acsaelm.4c01204 (PMC11500404; doi:10.1021/acsaelm.4c01204)
Supplement: Supplementary file 1 — el4c01204_si_001.pdf [file el4c01204_si_001.pdf]

Supporting Information

High performance MXene/$MnCo_2O_4$ supercapacitor device for powering small robotics

Nanasaheb M. Shinde and Martin Pumera*

Advanced Nanorobots & Multiscale Robotics Laboratory, Faculty of Electrical Engineering and Computer Science, VSB - Technical University of Ostrava, 17. listopadu 2172/15, 70800 Ostrava, Czech Republic

* Author for correspondence: M. Pumera, pumera.research@gmail.com

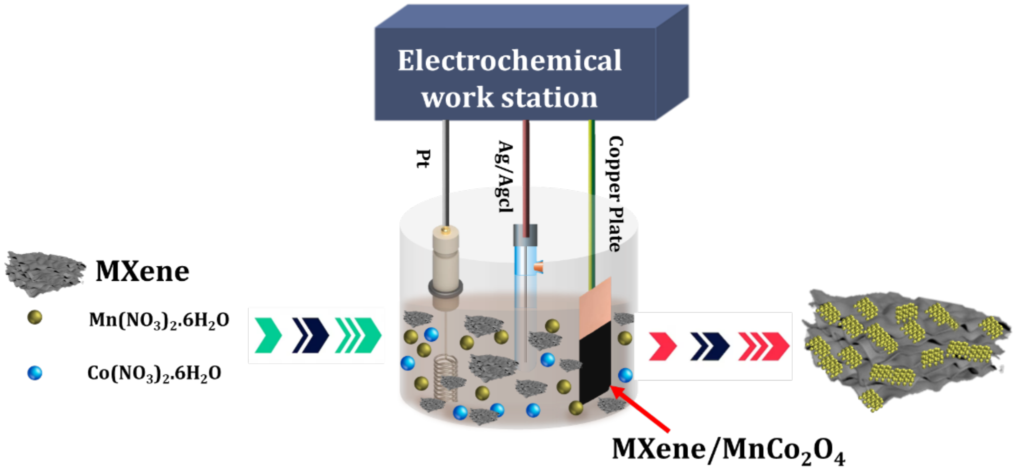

Scheme S1. Schematics of synthesis of MXene/$MnCo_2O_4$ nanocomposite.

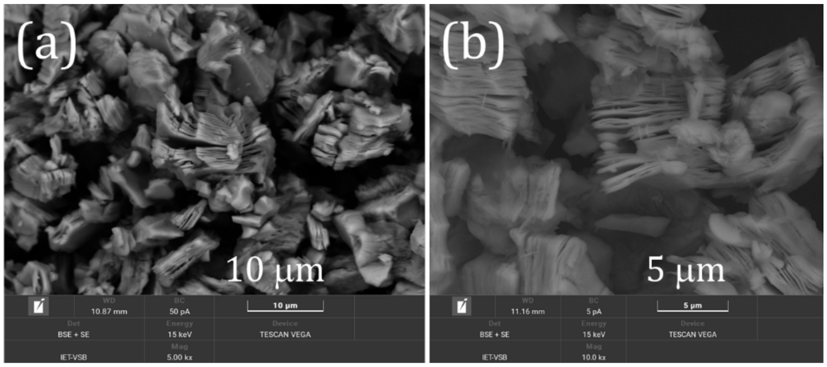

Figure S1. (a, b) FE-SEM images of MXene electrode.

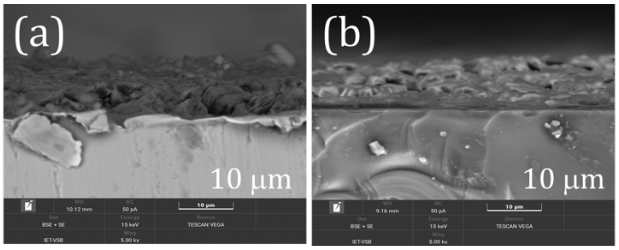

Figure S2. (a and b) FE-SEM cross section images of MXene/$MnCo_2O_4$ and MXene electrodes.

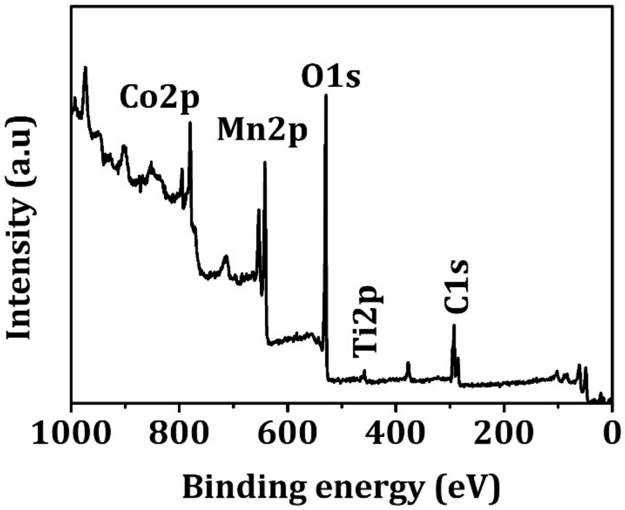

Figure S3. XPS survey of MXene/$MnCo_2O_4$ composite.

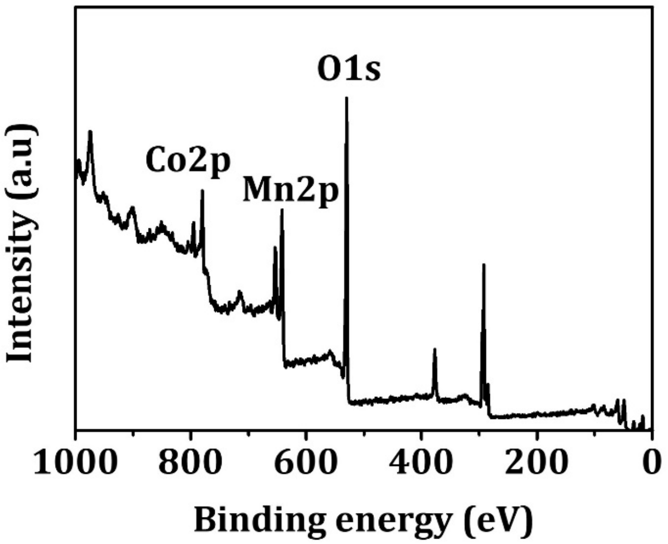

Figure S4. XPS survey of $MnCo_2O_4$.

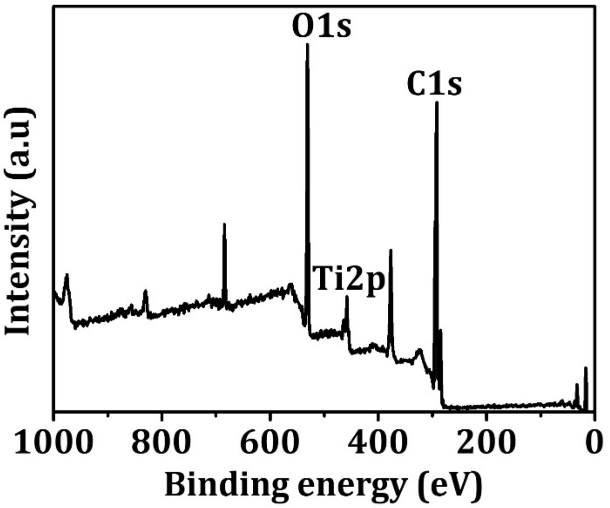

Figure S5. XPS survey of MXene.

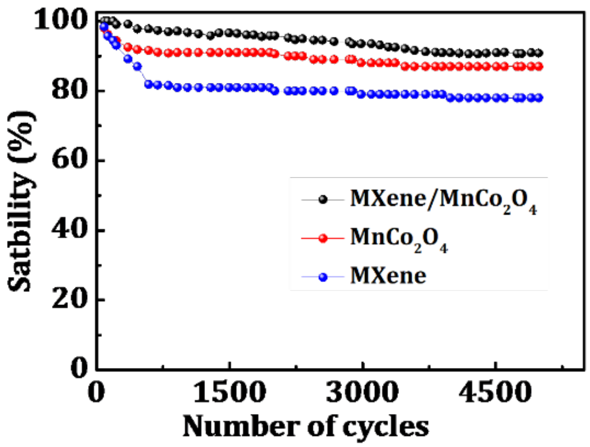

Figure S6. Stability of MXene/$MnCo_2O_4$, $MnCo_2O_4$ and MXene electrodes.

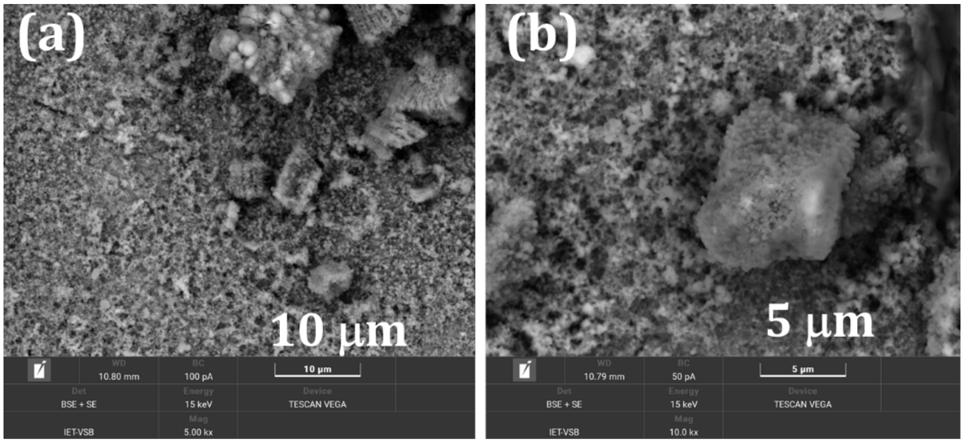

Figure S7. (a, b) FE-SEM images of MXene/$MnCo_2O_4$ electrode after 5000 cycles.

Synthesis of Bi_2O_3

The synthesis of Bi_2O_3 was performed with the help of a room temperature chemical bath deposition method. In short, 0.1 M bismuth nitrate $(BiNO_3)_3$ was mixed in 4 mL of nitric acid to get a clear and homogeneous solution; further, 50 mL of deionized water was added into it. Additionally, 0.5 mL of hydrochloric acid, as reducing agent, was commixed while continuously stirring. At the final stage, 500 μL of HCl was added into the above prepared solution. During precipitation, a heterogeneous reaction occurred that led to deposition of on copper substrate. The FE-SEM images scanned at different magnifications reveal [Figure. S8 (a-b)] a uniform coverage Bi_2O_3 (Figure. S8(a)). An average-size of the flower is ~3 μm. The higher magnified image (Figure. S8 (b)) conforms the existence of flower-like architecture which is composed of dozens of radially grown 2D nanoplatelets with each of ~0.1 μm thickness. As seen from FE-SEM cross -section image, the prepared Bi_2O_3 electrode thickness was ~11 μm [Figure. S8 (c)]. Additionally, the appearance of well reflected intense peaks (marked as '♠') different angle position was attributed to the presence of Bi_2O_3 (Figure. S8 (d)) [JCPDS card no 01-071-0467]. The cyclic voltammetry, galvanostatic charge discharge measurements were performed of Bi_2O_3. As seen from Figure S 9a and b, well define redox peaks were reflected, indicated that prepared Bi_2O_3 electrodes have pseudocapacitive. Figure S 9b, galvanostatic charge discharge measurements of a Bi_2O_3 electrode in 0 to −1 V potential range at different current densities i.e., from 1 to 4 $A.g^{-1}$. The Bi_2O_3 electrode demonstrates 166 − 49 $F.g^{-1}$ specific capacitance (Figure S 9c) and which remains about 90 % even after 5000 cycles (Figure S 9 d). Figure. S10 shows the FE−SEM images of Bi_2O_3 electrode after 5000 cycles, 3D flower-like of Bi_2O_3 electrode material was demolished after cycling process. Moreover, the flower-type Bi_2O_3 was distorted by forming agglomerated nanosheet which is eventually decreased stability of Bi_2O_3.

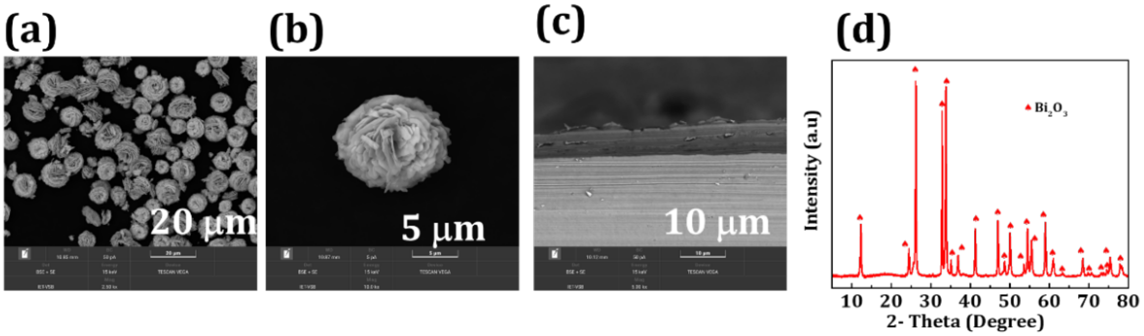

Figure S8. (a-b) FE-SEM, (c)FE-SEM cross section images and (d) XRD pattern of Bi_2O_3 electrode.

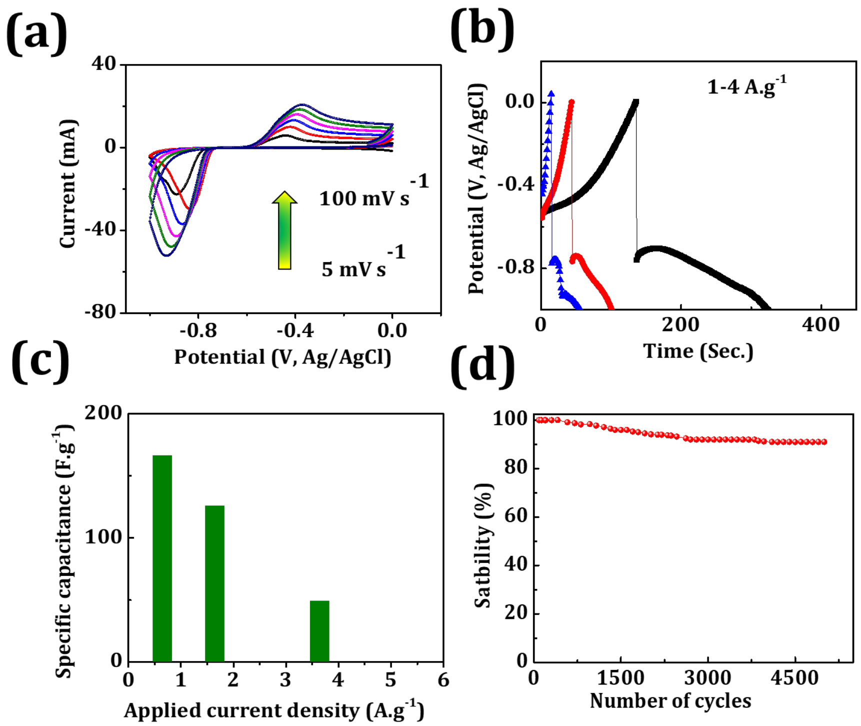

Figure S9. (a) cyclic voltammetry, (b) galvanostatic charge discharge curves and (c) specific capacitance versus applied current density and (d) stability of Bi_2O_3 electrode.

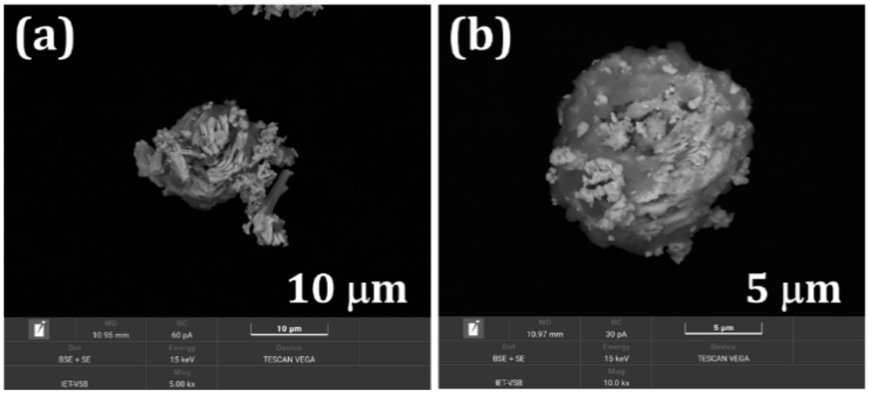

Figure S10. (a, b) FE-SEM images of Bi_2O_3 electrode after 5000 cycles.

S1. Materials Characterizations: Morphology study and MXene/ $MnCo_2O_4$ electrodes

X-ray diffraction (XRD, Rigaku Smart Lab for Cu–K radiation, =0.1541 nm) was performed to determine the crystallographic information and phase features of the product. For microscopic investigations of scanning electron microscope (SEM: Tescan Vega) with Tungsten cathode was used. Micrographs were obtained using combination of secondary electrons (SE) and backscattered electrons (BSE) mode (SE+BSE) and energy-dispersive X-ray spectroscopy (EDS: EDAX) with an acceleration voltage of 15 KeV. Samples before imaging were gold sputtered in order to ensure adequate electron conductivity. Fourier transform infrared (Thermo Scientific Nicolet iS10 FTIR Spectrometer) spectroscopy was used to characterize the functional groups. The measurements were carried out in the range 500−4000 cm^{-1} with a resolution of 2 cm^{-1} using transmission mode. The electrochemical characterizations of the manufactured electrodes, including cyclic voltammetry (CV), galvanostatic charge-discharge (GCD), and electrochemical impedance spectra (EIS), were investigated using an electrochemical workstation (Metrohm, Netherlands). A frequency range of 0.01 Hz to 100 kHz and a sinusoidal potential of 5 mV were used to study all electrodes with EIS at open circuit potential. GCD profiles were used to determine the stability test from gravimetric capacitance values.

S2. Formulae

The GCD curve-inspired SC of MXene, $MnCo_2O_4$ and MXene/$MnCo_2O_4$ electrodes were estimated in according as the following equation;

$$C = \frac{I\Delta t}{m\Delta V} \quad (1)$$

where, I is the discharge current, Δt and m are the discharge time and mass of active material; ΔV is the potential window.

Electrochemical quantities like energy density (*ED)* and power density (*PD)* were calculated by the following equations;

Energy density

$$ED = \frac{1}{2}C\Delta V^2 \quad (2)$$

Power density

$$PD = \frac{E}{\Delta t} \times 3600 \quad (3)$$

In this calculation formula, C is specific capacitance, ΔV is potential window (V), Δt is discharging time of prepared materials.

Table S1. Chemical composition of MXene/$MnCo_2O_4$ electrode.

**Element**	**Atomic Percentage**
Carbon	17.36%
Nitrogen	5.64%
Oxygen	44.43%
Aluminium	0.81%
Chlorine	1.56%
Titanium	5.51%
Manganese	7.04%
Cobalt	17.66%

Table S2. Half-cell electrochemical performance of MXene-based nanocomposite supercapacitor. Reference numbers point to main manuscript references.

Sr. No	Material	Synthesis Methods	Specific Capacitance	Stability/Cycles	Ref.
1	MXene/ $NiMoO_4$	Hydrothermal	1364 F.g^{-1}	68% /10000	[32]
2	MXene/Co_2NiO_4	Hydrothermal	719 F g^{-1}	83% /10000	[33]
3	MXene/ $MnCo_2O_4$	Electrodeposition	806 F g^{-1}	77%/3000	[34]
4	MXene/$CuMn_2O_4$	Hydrothermal	628 mF/cm^2	91%/10000	[35]
5	MXene/ Cu/Co	Molten	885 F g^{-1}	--	[36]
6	MXene/MnO_2	Refluxing	205 mFcm^{-2}	98%/10000	[50]
7	MXene/α-Fe_2O_3	Electrostatic	197 F g^{-1}	97%/2000	[51]
8	MXene/Co_3O_4	Hydrothermal	732 F g^{-1}	83%/5000	[52]
**9**	**MXene/$MnCo_2O_4$**	**Electrodeposition**	**668 F g^{-1}**	**90% /5000**	**Present work**

Table S3. Full-cell electrochemical performance of MXene-based supercapacitor device. Reference numbers point to main manuscript references.

Sr. No	supercapacitor device	Energy Density (Wh. Kg^{-1})	Power Density (W. Kg^{-1})	Stability/cycles	Ref.
1	MXene/ $RuCo_2O_4$// MXene/ $RuCo_2O_4$	14	22000	90%/5000	[31]
2	MXene/ $NiMoO_4$//rGH	33	400	72%/10000	[32]
3	MXene/Co_2NiO_4//AC	49	2752	94%/3500	[33]
4	MXene / $MnCo_2O_4$//AC	26	2880	93%/5000	[34]
5	MXene/ Co_3O_4//AC	26	700	81%/5000	[52]
6	MXene/polyaniline// MXene/polyaniline	31	1080	71%/4000	[53]
7	MXene/ Mn_3O_4/ Carbonized iron	28	463	92%/10000	[54]
8	MXene/ WO_3	22	1199	92%/5000	[55]
**9**	**MXene/$MnCo_2O_4$//Bi_2O_3**	**35**	**1854**	**94%/5000**	**Present work**
